# Preparation and ageing-resistant properties of polyester composites modified with functional nanoscale additives

**DOI:** 10.1186/1556-276X-9-215

**Published:** 2014-05-07

**Authors:** Gang Guo, Qiwu Shi, Yanbing Luo, Rangrang Fan, Liangxue Zhou, Zhiyong Qian, Jie Yu

**Affiliations:** 1 State Key Laboratory of Biotherapy and Cancer Center, West China Hospital, West China Medical School, Sichuan University, Chengdu 610065, People's Republic of China; 2 College of Physical Science and Technology, Sichuan University, Chengdu 610065, People's Republic of China; 3 History and Culture School, Sichuan University, Chengdu 610065, People's Republic of China; 4 National Engineering Research Center for Compounding and Modification of Polymer Materials, Guiyang 550014, People's Republic of China

**Keywords:** Nano-TiO_2_, Surface modification, Polyester composite, Crosslinking, UV ageing

## Abstract

This study investigated ageing-resistant properties of carboxyl-terminated polyester (polyethylene glycol terephthalate) composites modified with nanoscale titanium dioxide particles (nano-TiO_2_). The nano-TiO_2_ was pretreated by a dry coating method, with aluminate coupling agent as a functional grafting additive. The agglomeration resistance was evaluated, which exhibited significant improvement for the modified nanoparticles. Then, the effects of the modified nano-TiO_2_ on the crosslinking and ageing-resistant properties of the composites were studied. With a real-time Fourier transform infrared (FT-IR) measurement, the nano-TiO_2_ displayed promoting effect on the crosslinking of polyester resin with triglycidyl isocyanurate (TGIC) as crosslinking agent. Moreover, the gloss retention, colour aberration and the surface morphologies of the composites during accelerated UV ageing (1500 hours) were investigated. The results demonstrated much less degree of ageing degradation for the nanocomposites, indicating an important role of the nano-TiO_2_ in improving the ageing-resistant properties of synthetic polymer composites.

## Background

Ultra-violet (UV) radiation is a cytotoxic waveband of solar radiation reaching the Earth's surface [1]. Exposure to UV radiation from the sun is associated with different harmful effects for the organisms and synthetic polymer materials [2,3]. DNA is obviously one of the key targets for UV-induced damage in a variety of organisms which is traditionally attributed to the direct absorption of UV photons by nucleic acids and protein [1,4]. And the exposure of polymers to UV radiation may produce degradation discoloration and/or brittle fracture [5]. This is due to the UV irradiation-induced chemical reactions such as chain scission, crosslinking, oxidation or bond cleavage in the polymers [6-8]. All these damages may be undesirable due to their adverse impacts on the safety of organisms and the period of use of polymers. So, many organic and inorganic filters have been used to absorb and scatter UV radiation [3,9].

Titanium dioxide (TiO_2_), which can be either amorphous or crystalline [10], is used extensively in numerous applications, such as bone tissue engineering [11], bactericidal agents [12] and cosmetics [13]. The light absorption properties of anatase and rutile TiO_2_ are excellent since their absorption (approximately 400 nm) falls between the visible and UV regions [14]. Especially, ultrafine rutile TiO_2_ particles (< 100 nm) were used as a functional nanoscale additive because of its potential for the wide range (both UVB and UVA regions) of UV-ray shielding by their absorption, scattering and reflecting properties [15]. Once TiO_2_ is exposed to UV radiation, an electron is promoted from the valence band to the unoccupied conduction band, creating excitons [16]. Rayleigh's theory implies that shorter wavelengths of light are more efficiently scattered by smaller particles [17]. However, the smaller size leads to higher values of surface area which presents high surface energy and activity, so the nanoparticles tend to form agglomeration [18-20].

Particle aggregates in composite materials would decrease adhesion between nanoparticles and polymeric materials, which will result in an early failure at the interface and thus increase the susceptibility to physical and mechanical failure [21,22]. To achieve proper dispersion of nanoparticles in polymer matrix and to yield a better compatibility between the nanoparticles and polymeric materials, several groups have attempted to prevent the aggregation by modifying the surface groups of nano-TiO_2_ with different reagents including the silane coupling agent [23,24], the hydrolysis-condensation reactions (sol-gel method) [12] and *in situ* bulk polymerization [25,26]. Several polymers have been mixed with nano-TiO_2_ successfully including polystyrene (PS) [27], polythiophene (PTh) [28], poly(methyl methacrylate) (PMMA) [25], etc. Polyester resin has been widely studied as they possess many advantages including good mechanical properties, transparency, remarkable durability and flexibility [29,30]. However, the widespread use of polyester resin is blocked in many high-performance applications because of its limited ageing-resistant properties. Many methods have been used to improve the ageing-resistant properties of the polyester resin, such as synthesizing and modification of the resin, selection of curing system and curing agent in powder coatings and composited with suitable functional additives [31-34]. Nevertheless, to the best of our knowledge, it is still highly desirable to develop more industrial available processes for the surface modification of nano-TiO_2_, preparation of polyester/nano-TiO_2_, and their ageing-resistant properties.

In this investigation, we pretreated the nano-TiO_2_ particles and prepared the polyester/nano-TiO_2_ composites by melt-blend extrusion method. The aluminate coupling agent was employed as a functional grafting agent to realize a surface modification of the nano-TiO_2_. The particle size distribution, hydrophilic angle, UV reflection characteristic of the nano-TiO_2_, and its dispersion state in the polyester were detected. Moreover, the effect of nano-TiO_2_ on the gloss retention, colour aberration and morphology of the composites was investigated during the UV ageing. The dry modification method for the nano-TiO_2_ and its application as functional nanoscale additive are highly available for the widespread applications of polyester resin/TiO_2_ composites and would provide considerable insights into the protection of natural and synthetic carbohydrate polymers from the UV irradiation.

## Methods

### Materials

Carboxyl-terminated polyester resin (polyethylene glycol terephthalate) was purchased from Cytec Surface Specialties Inc., Woodland Park, NJ, USA, with an acid value of 33 mg KOH/g and a curing temperature of 190°C. Triglycidyl isocyanurate (TGIC) was used as curing agent and also purchased from Cytec Surface Specialties Inc. Rutile nano-TiO_2_ was purchased from Panzhihua Iron & Steel Research Institute in China, with grain size of 30 to 50 nm. Aluminate coupling agent was purchased from Chongqing Jiashitai Chemical Co. (Chongqing, China).

### Surface modification of nano-TiO_2_

The nano-TiO_2_ particles were modified with 1.5 wt.% aluminate coupling agent (based on the nano-TiO_2_ particles content). Firstly, the nano-TiO_2_ particles were put into a high-speed mixer (Dachen Machinery Manufacturing Co., Beijing, China, SHR-10A) and pre-mixed with a rotate speed of 2,000 rpm at 130°C. The collisions of the powder with stirring blade resulted in a high impaction and dispersion. Some powders were brought out for the other characterizations in this work. Then, 1.5 wt.% of aluminate coupling agent was added into the powder, and the mixtures were stirred further for 20 min. Subsequently, the mixtures were centrifuged and washed with fresh ethanol to remove the coupling agent adsorbed physically on the surface of nano-TiO_2_ particles. Finally, the modified particles were dried at 60°C for 2 h.

### Preparation of polyester/nano-TiO_2_ composites

We prepared the polyester/nano-TiO_2_ composite with different amounts of modified nano-TiO_2_. Firstly, four groups of the polyester resin 93 g and TGIC 7 g were blended physically and labelled as samples 1, 2, 3 and 4, respectively. Then, the modified nano-TiO_2_ with the amount of 0.5, 1.0, 1.5, and 2.0 wt.% based on the polyester resin content were added into the samples, respectively. The raw materials were mixed (at 90°C for 5 min) with a rotating speed of 2,000 rpm. During the mixing, the raw materials were melted and then extruded in a twin screw extruder. The extrudate was milled and sieved into particle with size less than 100 μm for further measurements.

The surface functional groups of nano-TiO_2_ were analyzed by Fourier transform infrared (FT-IR) spectrometer (Bruker, Tensor 27, Madison, WI, USA) with a detection resolution of 4 cm^-1^. The samples were acquired by compacting sheet of nano-TiO_2_/potassium bromide powder mixture (1:100 in mass) and then drying at 110°C for 5 min. The crystalline structure of the nano-TiO_2_ was detected by X-ray diffraction (XRD) (X'Pert, Philips, Amsterdam, The Netherlands) using a 4-kW monochromatic Cu Kα (*λ* = 0.15406 nm) radiation source.

The nano-TiO_2_ powder was pressed to be compact sheet, and then the surface modification effect of the samples was evaluated by measuring the hydrophilicity. An automatic contact angle analyzer (DSA 100, Kruss, Hamburg, Germany) was employed.

The nano-TiO_2_ powder was dispersed in ethanol with a viscosity of 0.5 mPa · S. Then, the particle size and size distribution of the nano-TiO_2_ powder was analyzed by Dynamic light scattering spectrum (DLS) (ZS-90, Malvern, Grovewood Road, Malvern, UK).

The dispersion of nano-TiO_2_ in the composites was investigated by field emission scanning electron microscopy (FE-SEM) (FEI, Inspect F, Hillsboro, OR, USA). Nano-TiO_2_ with 1.5 wt.% addition amount was added to prepare the composite powder, which was then cured in a PTFE mould at 190°C for 15 min and formed the sheets with thickness of 3 mm. Then, the sheets underwent brittle fracture in liquid nitrogen atmosphere, followed by gold sputter coated on the fracture sections. The FE-SEM was carried out with an accelerating voltage of 20 kV.

The reflection characteristics of the nano-TiO_2_ before and after surface modification were measured by ultraviolet-visible spectrophotometer (UV-vis) with a wavelength range from 190 to 700 nm.

The UV ageing resistance of the samples was carried out under the light-exposure conditions that simulate the requirements for real outdoor applications. A UV accelerated ageing chamber was equipped with fluorescent lamps emitting in the spectral region from 280 to 370 nm, of which the maximum irradiation peak occurs around 313 nm. The samples were placed for 1500 h in the chamber, and the time-dependent gloss retention and colour aberration of the samples across the ageing was measured.

The gloss retention (*G*_*r*_) was calculated according to formula (1):

(1) $$G_{r}=\frac{G_{0}‒G_{x}}{G_{0}}\times 100\%$$

*G*_*0*_ and *G*_*x*_ are the gloss of the sample without ageing and that of the sample aged with several hours, which were measured by a gloss spectrometer (WGG-66, Shanghai precision optical instrument Co., Ltd., Shanghai, China).

The colour aberration (Δ*E*) was calculated according to formula (2):

(2) $$\Delta E=\sqrt{\Delta {L_{x}}^{2}+\Delta {a_{x}}^{2}+\Delta {b_{x}}^{2}}$$

where *L*_*x*_, *a*_*x*_ and *b*_*x*_ are the lightness, redness-greeness and yellowness-blueness, respectively. These parameters of the samples before and after ageing were measured by a colour spectrometer (CR-10, Minolta Co., Osaka, Japan).

The surface morphology and roughness of the composites before and after ageing were studied by Atomic force microscopy (AFM) (Nanoscope Multimode APM, Vecco Instrument, Plainview, NY, USA) with a tapping mode under ambient condition.

## Results and discussion

**Figure 1:**
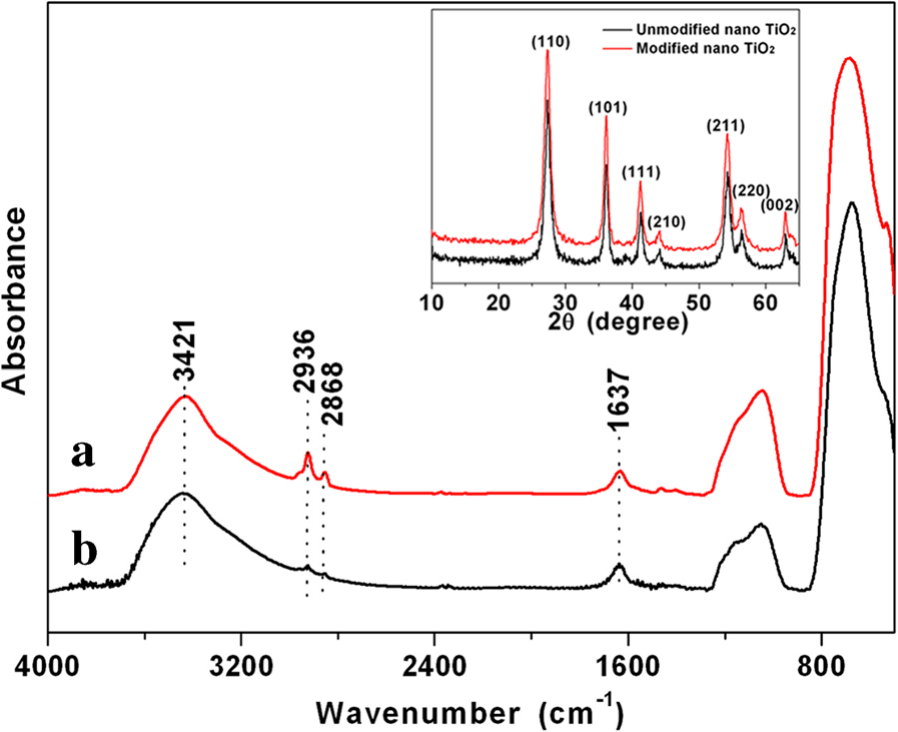
**FT-IR spectra of the nano-TiO**_**2**_**. (a) **Without modification and **(b) **modified with aluminate coupling agent. Inset, XRD patterns of the nano-TiO_2 _before and after the surface modification.

Figure 1 shows the FT-IR spectra of the unmodified nano-TiO_2_ and the modified nano-TiO_2_. The band around 3,421 and 1,637 cm^-1^ could be assigned to the hydroxyl groups on the surface of nano-TiO_2_. Compared with the spectrum of unmodified nano-TiO_2_, two absorbance peaks emerge around 2,936 and 2,868 cm^-1^ for the modified sample, which corresponds to the CH_2_ and CH_3_ stretching, respectively [15,35]. The result indicates that the organic functional groups were grafted to the nano-TiO_2_ during the surface modification. It is suggested that the hydroxyl groups on the surface of nano-TiO_2_ are active sites for the reaction with aluminate coupling agent [36,37]. Here, we detected the crystalline structure of the nano-TiO_2_ before and after the surface modification, and Figure 1 Inset shows that the sample stays in rutile phase in the experiments.

**Figure 2:**
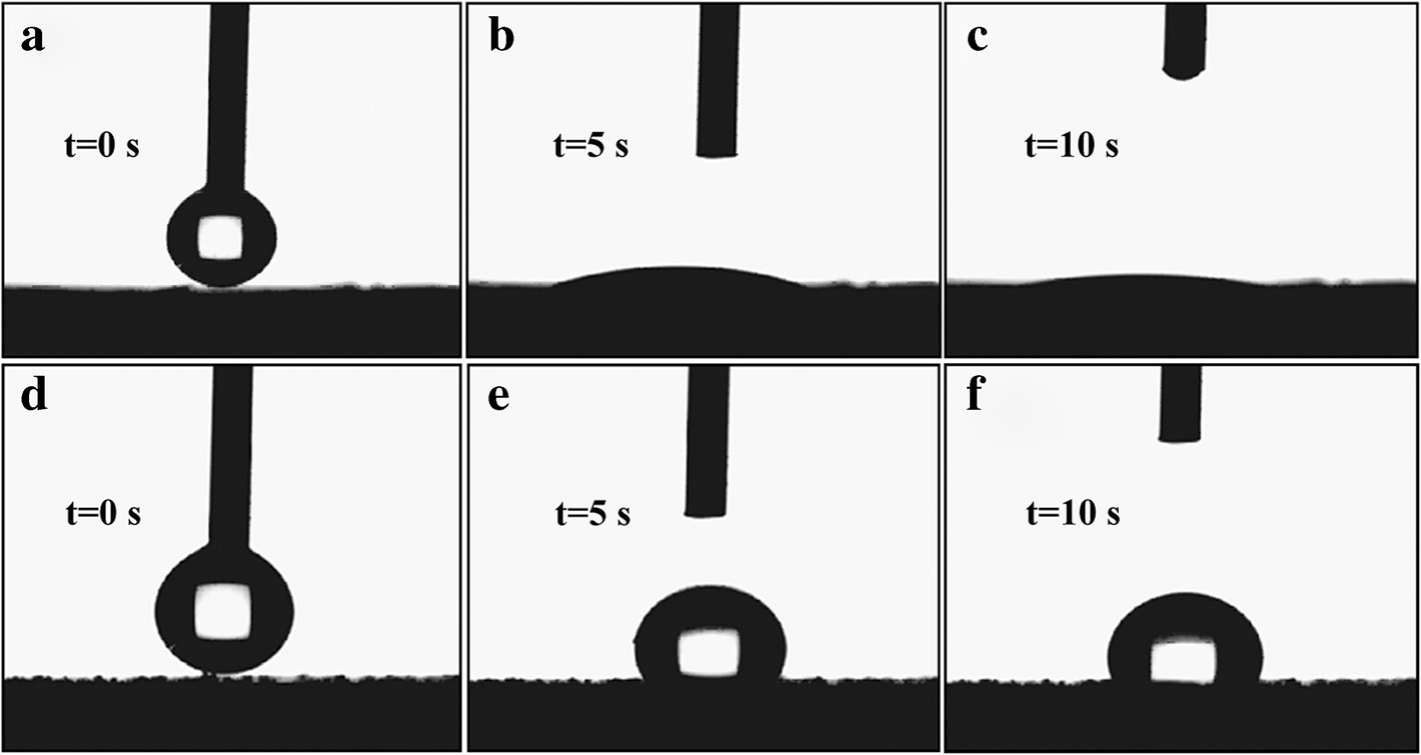
**Wetting and spreading images of the nano-TiO**_**2 **_**samples. (a to c)** Without modification and **(d to f)** modified with aluminate coupling agent.

The surface modification with coupling agent could graft organic groups to the nano-TiO_2_ particle and then transform its hydrophilic character to a hydrophobic character. We proved this effect by comparing the contact angle of the nano-TiO_2_ sheets before and after surface modification. As shown in Figure 2a,b,c, the DI water spreads on the sample without modification quickly, and the contact angle reduces to be nearly 0° after 10 s, indicating a well hydrophilicity for the nano-TiO_2_ without surface modification. It can be attributed to the high surface energy of the nano-TiO_2_. By contrast, the sample with modification shows a stable contact angle (Figure 2d,e,f). The value is still of about 90° when the contacting time is 10 s, which indicates a hydrophobic characteristic.

**Figure 3:**
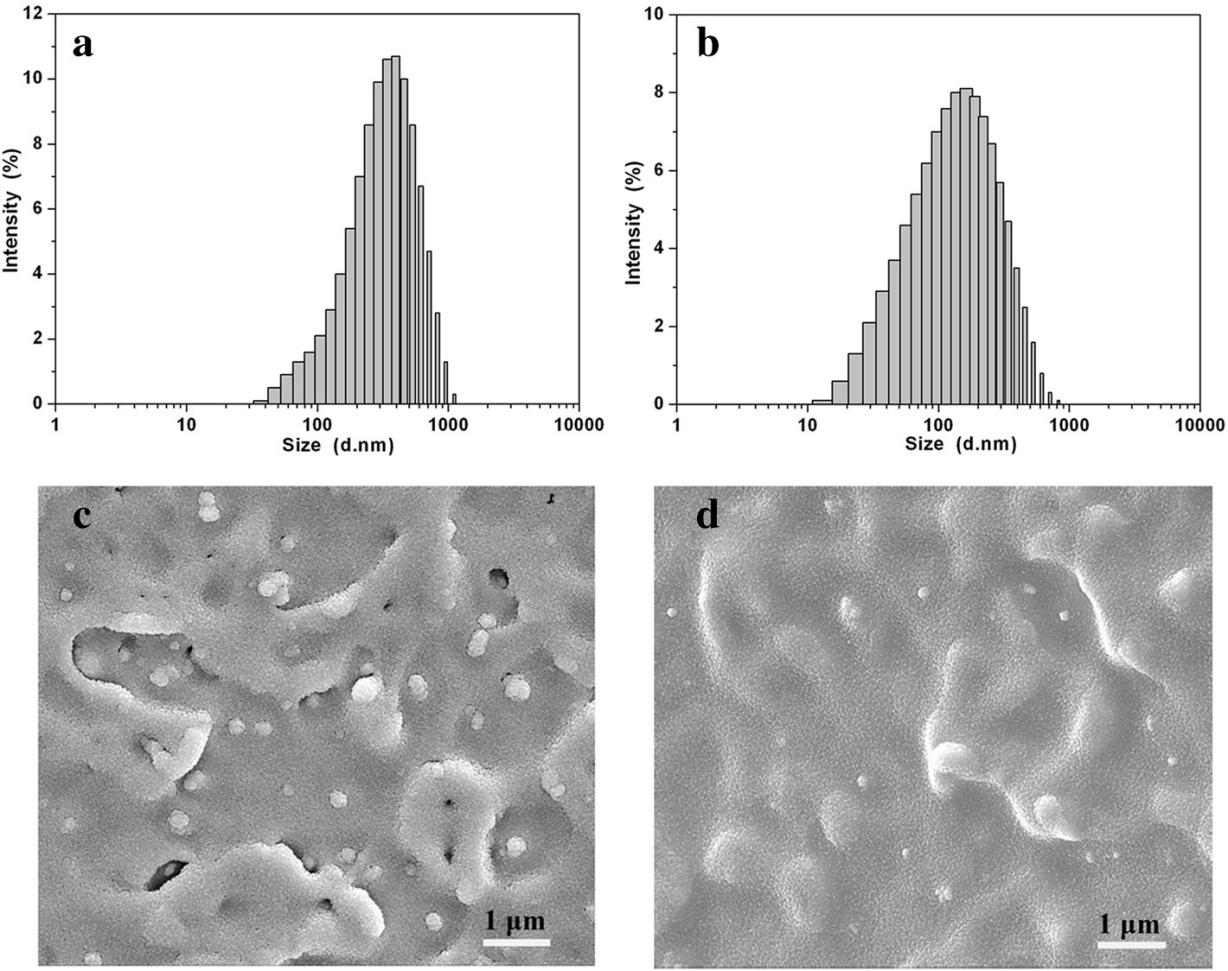
**Particle size distribution of the nano-TiO**_**2 **_**samples. (a)** Without modification and **(b)** modified with aluminate coupling agent; FE-SEM images of the polyester/nano-TiO_2_ composites: **(c)** the nano-TiO_2_ was not modified, and **(d)** the nano-TiO_2_ was modified with aluminate coupling agent.

Particle size distribution of the nano-TiO_2_ particles was determined by DLS. As shown in Figure 3a, the size distribution of the nano-TiO_2_ without modification mainly ranges from 200 to 600 nm, and the average particle size can be evaluated to be 303 nm. However, the modified particles exhibit the size distribution primarily ranging from 50 to 300 nm, with the average particle size of 129 nm (Figure 3b). The decreased average particle size indicates a lower agglomeration tendency resulted from the modification with aluminate coupling agent. The similar results for the surface modification of nano-TiO_2_ particles were also reported by Godnjavec et al. and Veronovski et al. [38,39].

Figure 3c,d compared the dispersion homogeneity of nano-TiO_2_ with 1.5 wt.% in the polymeric matrix. The unmodified nano-TiO_2_ agglomerated obviously, and the particle size was about 350 nm. It is resulted from limited compatibility of the unmodified nano-TiO_2_ with hydrophilic (Figure 3c). Nevertheless, Figure 3d exhibits fewer agglomerates of modified nano-TiO_2_ in the sample. Although the dispersion of nanoparticles is also limited due to the melt-blend extrusion, the size of the modified nano-TiO_2_ is uniform of about 100 nm. This is in accordance with the DLS result. Here, we could see significantly improved dispersion of nano-TiO_2_ particle in the polyester matrix, which further illustrates the importance of the surface modification process.

**Figure 4:**
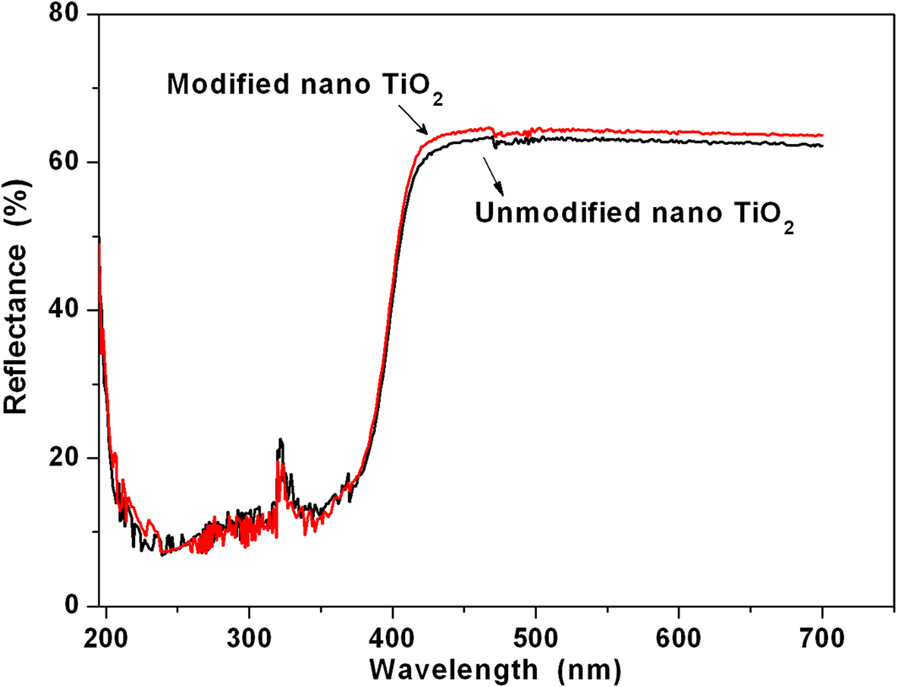
**UV-Vis reflection spectra of the nano-TiO**_**2 **_**samples. (a)** Without modification and **(b)** modified with aluminate coupling agent.

In addition, the effect of surface modification on the UV shielding ability of the nano-TiO_2_ particles was studied. Figure 4 presents the UV-vis reflection spectra of the nano-TiO_2_ before and after surface modification. The reflection of modified sample in the visible region (400 to 700 nm) is a little higher than that of the unmodified sample, which may be caused by the colour deviation in the modification process [38]. Furthermore, both of the UV reflection of the nano-TiO_2_ before and after surface modified are around 10% in the range of 190 to 400 nm, which is resulted primarily from the absorption and scattering of nano-TiO_2_[40]. This means that the nano-TiO_2_ exhibits excellent UV shielding ability and could protect the polymeric composites from UV degradation. Although the surface modification did not affect the UV shielding ability of the nano-TiO_2_, the UV shielding property of the polyester/nano-TiO_2_ composite is determined largely by the dispersion homogeneity of the nano-TiO_2_ powder. So, an increased uniformity in dispersion of nano-TiO_2_ in the polyester matrix will lead to larger amount of aggregated particle with smaller size in the matrix.

**Figure 5:**
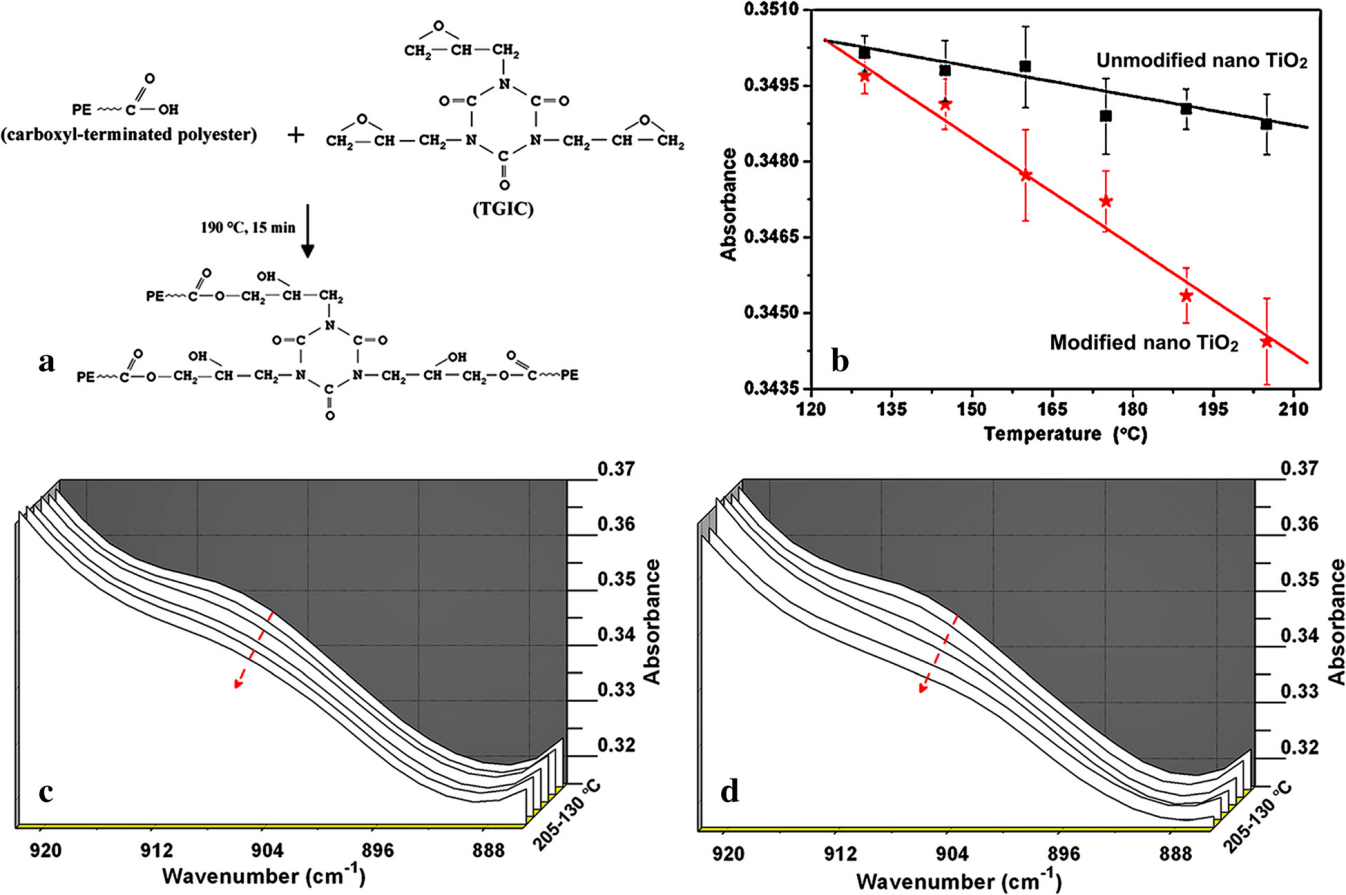
**Crosslinking through the reaction between the COOH of polyester and epoxy group of TGIC. (a)** Schematic mechanism for the crosslinking reaction between the polyester and TGIC; FT-IR spectra of the polyester/nano-TiO_2_ composites with 2.0 wt.% nano-TiO_2_ from 130°C to 205°C. **(b)** The nano-TiO_2_ was not modified. **(c)** The nano-TiO_2_ was modified with aluminate coupling agent. **(d)** The absorbance at 908 cm^-1^ as a function of temperature for the two systems.

We noticed that the carboxyl-terminated polyester could be used as a thermosetting resin with TGIC as crosslinking agent. The crosslinking takes place through the reaction between the COOH of polyester and epoxy group of TGIC [41]. The mechanism is described in Figure 5a. Several studies have reported that the hydroxyl groups can form ester bonds with carboxylic acid and promote the crosslinking [42,43]. Significantly, the modified nano-TiO_2_ is grafted with hydroxyl functional groups on the surface [44], which was also proved by the FT-IR spectra in Figure 1. Accordingly, the effect of modified nano-TiO_2_ on the crosslinking of polyester with TGIC was investigated by real-time FT-IR. We prepared the polyester/nano-TiO_2_ composites with unmodified and modified nano-TiO_2_ (the amount is 2.0 wt.%), and their FT-IR spectra were recorded from 130°C to 205°C.

Generally, the absorption band around 910 cm^-1^ was assigned to monitor the epoxy equivalent conversion (the C-O-C bond of epoxy groups) [45,46]. Figure 5b,c shows the FT-IR spectrum of the composites with unmodified and modified nano-TiO_2_, respectively. The decreased intensity of the absorption band could be attributed to the ring-opening of epoxy groups induced by the reaction between hydroxyl of COOH and epoxy groups during the crosslinking. In contrast to the sample with unmodified nano-TiO_2_, the sample with modified nano-TiO_2_ exhibits larger decreasing amplitude of the absorbance. Particularly, the absorbance at 908 cm^-1^ as a function of temperature for the two systems were plotted in Figure 5d, demonstrating a faster decreasing tendency of the absorbance at this band for the polyester/modified nano-TiO_2_ composite. It suggests a promoting effect of modified nano-TiO_2_ on the crosslinking reaction.

**Figure 6:**
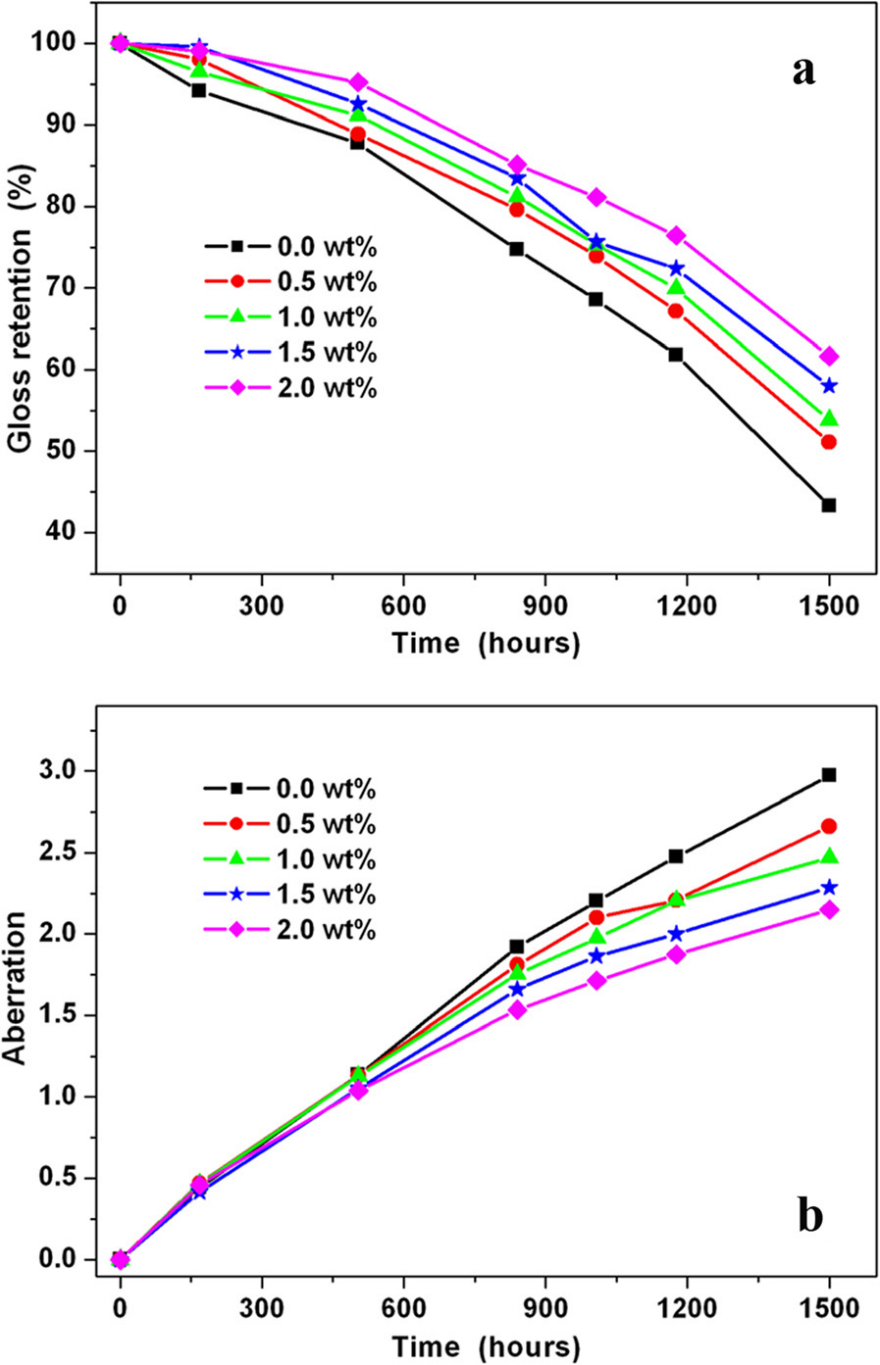
**Gloss retention (a) and colour aberration of the composites with different concentration of modified nano-TiO**_**2 **_**(b).** As a function of exposure times.

For the ageing resistance of the polyester/nano-TiO_2_ composites, gloss and colour aberration measurements were done during the exposure in the UV accelerated ageing chamber for 1500 h. In particular, the gloss changes and aberration are strongly correlated with the degradation level of the polymer composites. Figure 6a illustrates the gloss retention of the samples with different concentrations of modified nano-TiO_2_, as a function of exposure times. Compared with the sample without nano-TiO_2_, the gloss retention of the samples with nano-TiO_2_ improves significantly. In particular, the sample without nano-TiO_2_ exhibits gloss retention of 43.3%. By contrast, the gloss retention of the sample modified with 2.0 wt.% nano-TiO_2_ is 61.7%. So a 42.5% improvement was deduced. Furthermore, we noticed that the gloss retention of sample improves with the concentration of nano-TiO_2_ in the range 0.5 to 2.0 wt.%.

As a supplement, the colour aberration (determined by the value of Δ*E*) of the samples during the accelerated ageing was also observed. Figure 6b shows significant decrease in the colour aberration of the samples with modified nano-TiO_2_. This is due to lower degradation occurred in the polyester/nano-TiO_2_ composites. In this case, the nano-TiO_2_ plays a role in shielding UV radiation by absorption and scattering. After 1500-h ageing, the Δ*E* of the sample modified with 2.0 wt.% nano-TiO_2_ is 2.15, with reduction of 27.6% compared to a 2.97 Δ*E* of the sample without nano-TiO_2_. Coinciding with the results of gloss retention, the colour aberration of the sample decreases with the concentration of nano-TiO_2_.

**Figure 7:**
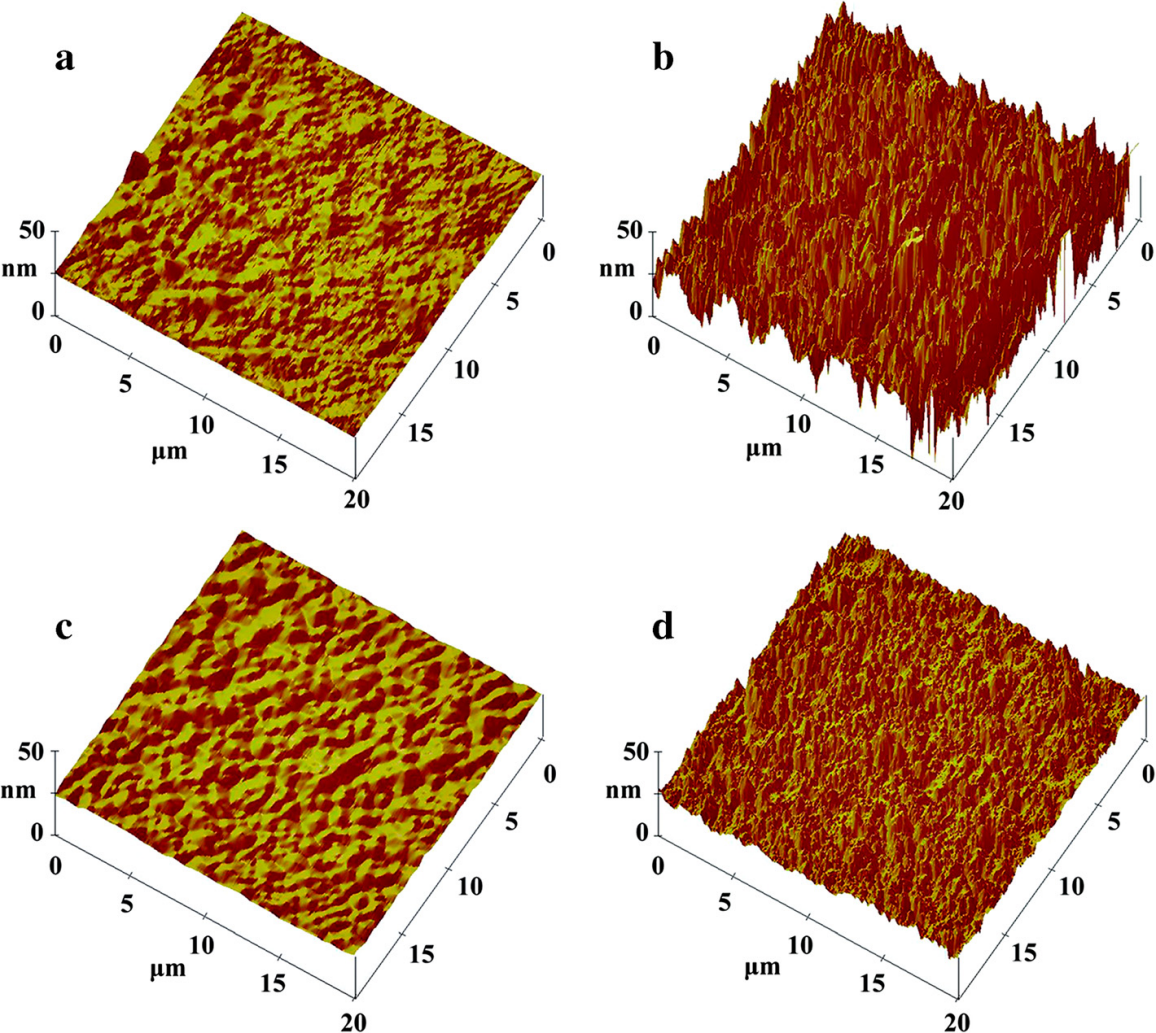
**Surface morphologies of composites before and after 1500-h UV ageing. (a)** and **(b)** without nano-TiO_2_; **(c)** and **(d)** with 2.0 wt.% modified nano-TiO_2_.

Figure 7 compares the surface morphologies of the sample without nano-TiO_2_ and the composite with 2.0 wt.% modified nano-TiO_2_, before and after 1500-h ageing. The scan size is 20 μm × 20 μm. Figure 7a,c shows that the samples are flat and compact before ageing. Nevertheless, the surface morphologies of the samples after ageing are quite different. The sample without nano-TiO_2_ presents rougher morphology with serious cracks and voids, suggesting obvious degradation due to the UV ageing (Figure 7b). By contrast, the polyester/nano-TiO_2_ composite exhibits a lower roughness significantly. Although some cracks emerge in the sample modified with nano-TiO_2_, its surface is still more compact than the sample without nano-TiO_2_ (Figure 7d). The mean value of surface roughness parameters (Ra) and root-mean-square (RMS) height of the samples were listed in Table 1. The differences in the surface morphologies of the sample before and after ageing are determined by the degradation extent across the ageing. It indicates that the nano-TiO_2_ plays an important role in improving the ageing-resistant property of the composites. The differences observed by AFM images are consistent with the results of gloss retention and colour aberration.

**Table 1:** Mean value of surface roughness parameters (Ra) and root-mean-square (RMS) height of the samples

**Samples**		**Ra/nm**	**RMS height/nm**
Polyester without nano-TiO_2_	0-h ageing	10.147	190.67
	1500-h ageing	145.22	2105.00
Polyester/2.0 wt% nano-TiO_2_ composite	0-h ageing	11.305	165.72
	1500-h ageing	49.534	523.00

Before and after 1500-h UV ageing.

## Conclusions

The nano-TiO_2_ was modified with aluminate coupling agent by a dry coating method. The FT-IR, contact angle and DLS measurements demonstrated a linkage of organic functional groups to the nano-TiO_2_, resulting in improved agglomeration resistance. Then, the modified nano-TiO_2_ was employed as a functional additive to prepare the polyester/nano-TiO_2_ composites by melt-blend extrusion method. With a real-time FT-IR study, the nano-TiO_2_ exhibited a promoting effect on the crosslinking reaction of polyester with TGIC. Furthermore, the gloss retention, colour aberration and the surface morphologies of the composites during an accelerated UV ageing were investigated. The results indicated that the nanocomposites exhibited much less degree of ageing degradation, due to a strong UV shielding ability of the nano-TiO_2_. Particularly, the polyester/nano-TiO_2_ presented an improvement of 42.5% in the gloss retention and a reduction of 27.6% in the colour aberration after 1500 h UV ageing. This work proposed a dry modification method for the nano-TiO_2_ and its application as functional nanoscale additive, which are highly available for the widespread applications of polyester resin/TiO_2_ composites, and would provide considerable insights into the protection of natural and synthetic carbohydrate polymers from the UV irradiation.

## Competing interests

The authors declare that they have no competing interests.

## Authors’ contributions

SQW carried out experimental work, analyzed the data and prepared the manuscript. GG participated in the analysis of the data and supervised the research work. YBL and RRF participated in experimental work. LXZ, ZYQ and JY participated in the studies, and improved the manuscript. All authors read and approved the final manuscript.
